# The relationship between physical activity, orthostatic blood pressure reactions and subclinical atherosclerosis: the Swedish CArdioPulmonary bioImage Study (SCAPIS)

**DOI:** 10.1038/s41371-025-01022-8

**Published:** 2025-05-05

**Authors:** Isabella Kharraziha, Ensieh Memarian, Örjan Ekblom, Anders Gottsäter, Gunnar Engström, Viktor Hamrefors

**Affiliations:** 1 Department of Clinical Sciences, Lund University, Malmö, Sweden; 2 Department of Cardiology, Skåne University Hospital, Malmö, Sweden; 3 Department of Internal Medicine, Skåne University Hospital, Malmö, Sweden; 4 Department of Physical Activity and Health, The Swedish School of Sport and Health Sciences, Stockholm, Sweden; 5 Department of Neurobiology, Care Sciences and Society, Division of Nursing, Karolinska Institutet, Stockholm, Sweden

**Keywords:** Risk factors, Hypertension, Coronary artery disease and stable angina

## Abstract

An abnormal blood pressure (BP) response on standing is associated with atherosclerotic cardiovascular disease (CVD). The role of physical activity (PA) on orthostatic BP-reactions and its relation to subclinical atherosclerosis is unclear. We aimed to assess the association between PA and orthostatic BP-reactions, and whether PA modifies the relationship between orthostatic BP-reactions and subclinical atherosclerosis. A total of 5,396 middle aged subjects from the population-based SCAPIS-study were included. Associations between orthostatic BP-response and accelerometer-derived PA were studied using linear regression. Interaction analyses were performed to study modifying effects of PA on the relationship between orthostatic BP-response and subclinical coronary atherosclerosis, assessed by coronary artery calcium score (CACS). Moderate to vigorous PA (MVPA) was associated with less pronounced orthostatic systolic BP (SBP) increase but more pronounced orthostatic diastolic BP increase after adjusting for age, sex, total wear time, proportion weekend days and season (Beta per 1%-increase(mmHg):0.12; *p* = <0.01 and −0.06; *p* = 0.02, respectively). Subjects with high MVPA were less likely to have orthostatic hypertension (OHTN), but more likely to have orthostatic hypotension (OH; *p* = 0.002 for both). Individuals with higher CACS were more likely to have OH (*p* = 0.041) but not OHTN (*p* = 0.276). There were no interactions of PA on the association between orthostatic BP-response and CACS. In conclusion, physically active middle-aged individuals are less likely to show inappropriate SBP-increase upon standing, but more likely to have excessive SBP-decrease. PA does not modify the association between orthostatic BP-response and subclinical atherosclerosis. The relationship between PA, orthostatic BP and CVD is likely to be complex.

## Introduction

When standing, blood pressure (BP) and heart rate (HR) control is challenged by gravitational forces. Compensatory mechanisms mediated by the autonomic nervous system maintains postural BP homeostasis. Impairment of the compensatory response to orthostasis may lead to a fall in BP, known as orthostatic hypotension (OH) [1]. In addition to possible symptoms of orthostatic intolerance, OH is associated with increased mortality and cardiovascular disease (CVD), such as stroke, heart failure, atrial fibrillation, and myocardial infarction [2], as well as subclinical atherosclerotic CVD [3].

In contrast to OH, orthostatic hypertension (OHTN), defined as increased BP from supine to upright position [4], has attracted less attention. Still, OHTN appears to predict arterial hypertension later in life and has been associated with an increased cardiovascular risk [5]. To date, there is no consensus regarding the definition of OHTN, making it difficult to assess its true prevalence [4]. Since both OH and OHTN herald increased risk of CVD, it seems reasonable to screen patients for abnormal orthostatic BP reactions and find strategies to lower the risk of cardiovascular complications.

Physical activity (PA) reduces the risk of CVD [6], and is often recommended to patients to improve modifiable risk factors, such as low concentrations of high-density lipoprotein (HDL) cholesterol, excess adiposity, high BP, and glucose metabolism [7]. In addition, PA may improve cardiovascular autonomic function [6]. However, the association between exercise and orthostatic BP is still quite unclear.

We hypothesised that higher levels of physical activity are associated with improved autonomic function, leading to more stable orthostatic BP compared to a sedentary lifestyle. Given the links between impaired orthostatic BP responses and cardiovascular disease, we further hypothesised that physical activity may modify the relationship between orthostatic BP changes and subclinical atherosclerosis. To test this, we examined the association between accelerometer-based PA and orthostatic BP changes and explored whether PA modifies the relationship between orthostatic reaction and subclinical atherosclerosis.

## Methods

### Study population

The Swedish CArdioPulmonary bioImage Study, SCAPIS [8], is a population-based cohort for the study of CVD and chronic obstructive pulmonary disease. Between 2013 and 2018, a total of 30 154 men and women, aged 50–64 years, were randomly selected and examined at six Swedish university hospitals as previously described [8]. The subjects received an invitation letter and the only exclusion criteria was inability to understand written and spoken Swedish [8].

**Fig. 1:**
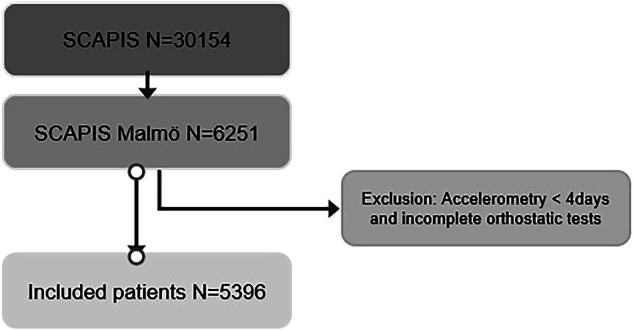
Study Population. Study population flow chart. Abbreviations: SCAPIS = Swedish CArdioPulmonary bioImage Study. SCAPIS is a population-based cohort for the study of cardiovascular disease and chronic obstructive pulmonary disease. Between 2014 and 2018, a total of 30 154 men and women, aged 50–64 years, were randomly selected and investigated at six Swedish university hospitals (Gothenburg, Linköping, Malmö/Lund, Stockholm, Umeå, and Uppsala). Orthostatic BP test was part of the examination at the Malmö site and the current study is therefore based on the SCAPIS-Malmö subcohort (*n* = 6 251). After excluding patients with incomplete orthostatic tests and accelerometer wear-time less than four days, a total of 5 396 individuals remained and were included to the current study.

Orthostatic BP test was part of the examination in Malmö, why the current study was based on the SCAPIS-Malmö subcohort (n = 6 251). The participation rate was 53%. A total of 5 846 individuals performed accelerometry, of which 5 588 wore the accelerometer for a minimum of 600 min/day, during four days. After excluding patients with incomplete orthostatic tests and accelerometer wear-time less than four days, a total of 5 396 individuals remained and were included to the study. A flow-chart on study population is shown in Fig. 1. The sample size was maximized from available data of our cohort (the SCAPIS-Malmö cohort) and is approximately equal to that of our previous work [9, 10].

### Accelerometery

Accelerometer assessed sedentary time and PA patterns were derived from tri-axial accelerometers, ActiGraph model wGT3X-BT, ActiGraph LCC, Pensacola, FL, USA. As previously described [11], participants wore the accelerometers for 7 days and were instructed to wear the accelerometer in an elastic belt over the right hip during all waking hours, except during water-based activities. ActiLife v.6.13.3 software was used to initialize the accelerometers and to transfer and process the collected data. Raw accelerometer data, recorded from three axes, were combined into a resulting vector, expressed in counts per minute (cpm), collected as 60-second periods using a low-frequency extension filter. Average cpm was calculated as total vector magnitude cpm divided by minutes of wear time. In the current study, we chose to use cut-offs for sedentary behaviour and PA patterns from previous studies to enable comparisons. Sedentary time was defined as <200 cpm [12], moderate intensity PA (MPA) as 2 690–6 166 cpm, vigorous PA (VPA) as ≥6 167 cpm [13], and moderate to vigorous PA (MVPA) as ≥2 690. The PA pattern is presented as percentage of wear time spent in different intensity-specific categories. In addition, a high daily sedentary time was defined as 9.5 h/day, which we translated into percentage of hypothetical 16 h of waking time (9.5 h/16.0 h = 59.4%). As previously described [11], a potential “at-risk” joint behaviour of sedentary and VPA, as well as sedentary and MPA, was defined as a high daily sedentary time (>9.5 h/day) and low VPA (<75 min/week) or low MPA (<150 min/week), respectively.

### Orthostatic tests

The orthostatic tests were conducted using standardized measurement protocols from SCAPIS, which have been applied in previous studies [9, 10]. After ten minutes of rest, supine BP was measured twice in the non-dominant arm (measurements were separated by one minute). After three minutes of active standing, BP was measured twice immediately after each other. Mean systolic BP (SBP) and mean diastolic BP (DBP) was calculated in both supine position and after 3 min of standing. An Omron M10-IT automatic BP reader was used.

### Coronary artery calcium score

Coronary artery calcium score (CACS) is a marker of subclinical coronary atherosclerosis and a risk marker for future CVD [14]. Cardiac imaging in SCAPIS has been described in detail previously [8]. Briefly, CACS was assessed using a dedicated dual source CT scanner equipped with a Stellar Detector (Somatom Definition Flash, Siemens Medical Solutions, Forchheim, Germany). The calcium content (>130 HU) in each coronary artery was measured and summed to produce CACS according to Agatston [15]. Subjects with implanted stent or post coronary artery bypass graft were not evaluated for CACS.

### Included variables and statistical analysis

Delta SBP and delta DBP were defined as [mean SBP/DBP supine-mean SBP/DBP at 3 min of standing], meaning that a positive value corresponded to a decrease in SBP or DBP. OH was defined as a decrease in SBP from baseline ≥20 mmHg or DBP ≥ 10 mmHg, or a decrease in systolic BP to <90 mmHg [1]. OHTN was defined as an increase in SBP from supine to ≥20 mmHg, as commonly determined in previous studies [16]. Information on smoking habits and hypertension diagnosis were derived from subject questionnaires. Current smoking was defined as self-reported current or occasional smoker. Diabetes/prediabetes was defined as either impaired fasting glucose ≥6.1 mmol/l and <7.0 mmol/l, elevated HbA1c > 42 mmol/mol and <48 mmol/mol, diabetes according to questionnaire or fasting *p*-glucose ≥7.0 mmol/l day 1 or HbA1c ≥ 48 mmol/mol.

Normality was assumed for all continuous data, except CACS, after visual inspection of distribution curves. Group differences between quartiles of sedentary PA or MVPA were compared using a one-way ANOVA after testing for the homogeneity of variance. If the homogeneity of variance had a significance level of <0.05, a Welch test was conducted instead. Independent samples t-test was used to study group differences between participants “at risk” and those not “at risk”. For non-parametric data, Mann-Whitney or Kruskal-Wallis test was performed. For dichotomous data, Pearson’s chi2-test was used.

Multivariable-adjusted linear regression models were used to study the association between PA and BP, adjusting for age, sex, diabetes, smoking, pulse, total accelerometer wear time, proportion of weekend days, and season. Total wear time was defined as total minutes with activity data. To adjust for seasonal variation, three dummy variables were created (winter, summer, spring), with fall as reference category.

### Interaction analyses

Logistic regression or linear regression was performed to study potential interaction effects from PA on the association between orthostatic BP reaction and subclinical atherosclerosis. In case the correlation coefficient was r > 0.7, variables were mean-centred to reduce the risk of multicollinearity, which has been suggested as an appropriate threshold previously [17]. In the logistic interaction analyses, OH, OHTN or CACS > 0 were the dependent variables, whereas Δ SBP was the dependent variable in the linear regression analyses. Either sedentary time, MVPA, “at risk”, CACS > 0, OH, OHTN or ΔSBP were entered as independent variables in addition to the interaction terms sedentary time*CACS > 0, sedentary time*OH, sedentary time*ΔSBP, sedentary time*OHTN, MVPA*CACS > 0, MVPA*OH, MVPA*ΔSBP, MVPA*OHTN, “at risk”*CACS > 0, “at risk”*OH, “at risk”*ΔSBP, “at risk”*OHTN (multiplicative).

The data were analysed using IBM SPSS Statistics for Windows, version 28 (IBM Corp., Armonk, N.Y., USA). A two-sided *p*-value of <0.05 was considered significant for all tests.

## Results

### Baseline characteristics

Participants’ mean age was 57.5 ± 4.3 years, with 54.1% women. A total of 123 subjects met criteria for OH, whereas a total of 303 subjects met criteria for OHTN. Baseline characteristics stratified by hypertension vs. no hypertension, diabetes/prediabetes vs. no diabetes/prediabetes, and sex are presented in Tables 1, 2, and supplementary table 1, respectively.

**Table 1:** Baseline Characteristics of Hypertensive and Non-Hypertensive middle-aged men and women.

Characteristic (total n = 5221)	Hypertensive (n = 1269)	Non-hypertensive (n = 3952)	p-value
Age (years)	58.7 ± 4.2	57.2 ± 4.3	<0.001
Women (%)	23.2	76.8	0.047†
Men (%)	25.6	74.4	†
Current smoker (%)	16.3	16.7	0.791*
Diabetes/prediabetes (%)	34.8	16.5	<0.001**
Supine HR (beats/minute)	62.4 ± 9.4	60.4 ± 8.8	<0.001
Supine SBP (mmHg)	131.4 ± 16.3	120.1 ± 15.7	<0.001
Supine DBP (mmHg)	81.2 ± 9.3	75.1 ± 9.4	<0.001
ΔSBP (mmHg)	−4.3 ± 11.3	−3.7 ± 10.0	0.100
ΔDBP (mmHg)	−9.2 ± 7.0	−9.5 ± 6.3	0.070
CACS (median (Q1-Q3))	4 (0–78)	0 (0–17)	<0.001***††

Continuous variables are expressed as mean ± standard deviation, except for coronary artery calcium score which is expressed as median (Q1- Q3). Dichotomous data are presented as percentages within each group. P-values denote overall differences from independent samples t-test for continuous data and Pearson’s chi2-test for dichotomous data unless otherwise indicated.

Missing data: *=45, **=2, ***=159. *Q* Quartile, *HR* heart rate, *SBP* systolic blood pressure, *DBP* diastolic blood pressure, *CACS* coronary artery calcium score, *bpm* beats per minute.

†The p-value represents the difference in the distribution of hypertension between males and females, calculated using Pearson’s chi2-test.

†† p-value from Mann-Whitney U test.

**Table 2:** Baseline characteristics of participants with and without diabetes or prediabetes.

Characteristic (total n = 5393)	Diabetes or prediabetes (n = 1141)	No diabetes or prediabetes (n = 4252)	p-value
Age (years)	58.4 ± 4.2	57.3 ± 4.3	<0.001
Women (%)	17.7	82.3	<0.001†
Men (%)	25.2	74.8	
Current smoker (%)	19.1	16.1	0.016*
Hypertension (%)	40.4	20.0	<0.001**
Supine HR (beats/minute)	63.3 ± 9.6	60.2 ± 8.7	<0.001
Supine SBP (mmHg)	126.7 ± 16.1	121.7 ± 16.4	<0.001
Supine DBP (mmHg)	78.1 ± 9.5	76.1 ± 9.7	<0.001
ΔSBP (mmHg)	−4.2 ± 11.1	−3.7 ± 10.1	0.184
ΔDBP (mmHg)	−8.7 ± 6.6	−9.7 ± 6.4	<0.001
CACS (median(Q1-Q3))	2 (0–72)	0 (0–21)	<0.001††***

Continuous variables are expressed as mean ± standard deviation, except for coronary artery calcium score which is expressed as median (Q1- Q3). Dichotomous data are presented as percentages within each group. P-values denote overall differences from independent samples t-test for continuous data and Pearson’s chi2-test for dichotomous data unless otherwise indicated.

Missing data: *=138, **=174, ***=170. *Q* Quartile, *HR* heart rate, *SBP* systolic blood pressure, *DBP* diastolic blood pressure, *CACS* coronary artery calcium score, *bpm* beats per minute.

†The p-value represents the difference in the distribution of hypertension between males and females, calculated using Pearson’s chi2-test.

†† p-value from Mann-Whitney U test.

High proportion of sedentary time was associated with higher BMI, LDL, higher proportion of hypertension, diabetes, CACS, supine SBP and DBP, whereas no significant difference was found in delta SBP or delta DBP. Details on baseline characteristics and orthostatic blood pressure according to sedentary level, MVPA, and “at risk” individuals are shown in Tables 3–5-respectively.

**Table 3:** Baseline characteristics according to % sedentary time among middle aged men and women.

	% sedentary time				
	Lowest			Highest	p-value
TOTAL: 5 396	Q1 (n = 1 494)	Q2 (n = 1 323)	Q3 (n = 1 338)	Q4 (n = 1 241)	
% sedentary time	39.7 ± 5.6	50.0 ± 2.0	57.0 ± 2.0	66.1 ± 4.6	<0.001^a^
Age (years)	57.4 ± 4.3	57.5 ± 4.3	57.6 ± 4.3	57.7 ± 4.3	0.542
Women (%)	65.1	56.7	51.9	40.4	<0.001
Supine SBP (mmHg)	122.0 ± 16.9	121.6 ± 15.8	122.8 ± 16.2	124.7 ± 16.8	<0.001
Supine DBP (mmHg)	75.8 ± 9.8	75.9 ± 9.4	76.7 ± 9.6	77.9 ± 9.8	<0.001
ΔSBP (mmHg)	−3.4 ± 10.0	−3.7 ± 10.4	−4.0 ± 10.2	−4.2 ± 10.6	0.145
ΔDBP (mmHg)	−9.4 ± 6.4	−9.8 ± 6.4	−9.4 ± 6.5	−9.4 ± 6.6	0.291
Supine HR (bpm)	60.4 ± 8.4	60.3 ± 9.1	61.0 ± 9.0	62.0 ± 9.6a	<0.001^a^
Orthostatic hypotension (%)	2.5	2.6	1.8	2.2	0.469
Orthostatic hypertension (%)	4.4	5.8	5.9	6.5	0.098
Current smoking (%)	17.2	16.2	15.9	17.5	0.676
Hypertension (%)	19.5	22.1	24.1	32.6	<0.001
Diabetes/prediabetes (%)	18.2a	20.6	21.0b	25.5	<0.001
BMI (kg/m2)	26.1 ± 4.2	26.7 ± 4.0	27.3 ± 4.4	28.7 ± 5.1	<0.001^a^
LDL (mmol/l)	3.5 ± 0.9c	3.6 ± 0.9 d	3.6 ± 1.0 f	3.6 ± 1.0e	0.015^a^
CACS (median [Q1-Q3])	0 (0–22.0)	0 (0–22.0)	0 (0–29.0)	1 (0–42.0)	<0.001^b^
CACS > 0 (%)	41.2	40.3	44.0	50.3	<0.001

Continuous variables are expressed as mean ± standard deviation, except for coronary artery calcium score which is expressed as median (Q1- Q3). Dichotomous data are expressed as percentages of total within each group. P-values denote overall differences from one-way ANOVA for continuous data and Pearson’s chi2-test for dichotomous data unless otherwise indicated. Missing data: a=1, b=2, c=3, d=4, e=7, f=8. Data on smoking habits, hypertension and coronary artery calcium score were missing in 139, 175 and 170 participants respectively. *Q* Quartile, *HR* heart rate, *SBP* systolic blood pressure, *DBP* diastolic blood pressure, *BMI* body mass index, *LDL* low-density lipoprotein, *CACS* coronary artery calcium score, *bpm* beats per minute.

^a^=Welch test.

^b^=Kruskal-Wallis test.

**Table 4:** Baseline characteristics according to % moderate-vigorous physical activity among middle aged men and women.

	%moderate-vigorous physical activity				
	Highest			Lowest	p-value
TOTAL: 5 396	Q4 (n = 1 159)	Q3 (n = 1 045)	Q2 (n = 1 358)	Q1 (n = 1 834)	
%moderate-vigorous PA	11.4 ± 3.0	7.5 ± 5.0	5.5 ± 5.0	3.0 ± 9.8	<0.001^a^
Age (years)	57.0 ± 4.3	57.3 ± 4.2	57.5 ± 4.3	58.0 ± 4.2	<0.001
Women (%)	46.4	51.5	57.8	57.6	<0.001
Supine SBP (mmHg)	121.6 ± 16.0	121.4 ± 15.5	122.8 ± 16.7	124.1 ± 17.0	<0.001^a^
Supine DBP (mmHg)	75.8 ± 9.4	75.8 ± 9.3	76.5 ± 10.0	77.4 ± 9.9	<0.001^a^
ΔSBP (mmHg)	−3.3 ± 10.3	−3.1 ± 10.1	−3.9 ± 10.1	−4.5 ± 10.6	0.001
ΔDBP (mmHg)	−9.8 ± 6.6	−9.7 ± 6.0	−9.6 ± 6.4	−9.1 ± 6.6	0.032^a^
Supine HR (beats/minute)	59.3 ± 8.5	60.0 ± 9.0	60.6 ± 8.8	62.7 ± 9.3a	<0.001
Orthostatic hypotension (%)	3.5	2.5	2.2	1.4	0.002
Orthostatic hypertension (%)	4.6	4.5	5.2	7.3	0.002
Current smoking (%)	12.1	13.2	15.4	22.5	<0.001
Hypertension (%)	21.3	22.0	24.6	27.3	<0.001
Diabetes/prediabetes (%)	19.0b	18.6	20.2	24.7a	<0.001
BMI (kg/m2)	26.7 ± 4.4	26.8 ± 4.2	26.9 ± 4.4	27.7 ± 4.8	<0.001^a^
LDL (mmol/l)	3.6 ± 0.9e	3.5 ± 0.9a	3.6 ± 0.9 d	3.6 ± 1.0c	0.267
CACS (median [Q1-Q3])	0 (0–27.8)	0 (0–23.5)	0 (0–24.0)	0 (0–36.0)	0.014^b^
CACS > 0 (%)	43.4	40.9	43.0	46.1	0.055

Continuous variables are expressed as mean ± standard deviation, except for coronary artery calcium score which is expressed as median (Q1- Q3). Dichotomous data are expressed as percentages of total within each group. P-values denote overall differences from one-way ANOVA for continuous data and Pearson’s chi2-test for dichotomous data unless otherwise indicated. Missing data: a=1, b=2, c=10, d=5, e=6. Data on smoking habits, hypertension and coronary artery score were missing in 139, 175 and 170 participants respectively. *Q* Quartile, *HR* heart rate, *SBP* systolic blood pressure, *DBP* diastolic blood pressure, *BMI* body mass index, *LDL* low-density lipoprotein, *CACS* coronary artery calcium score.

^a^=Welch test.

^b^=Kruskal-Wallis test.

**Table 5:** Baseline characteristics according to individuals “at risk” among middle aged men and women.

	Low risk	Individuals at risk	p-value
TOTAL: 5 396	N = 3 919	N = 1 477	
Average counts per minute	760.4 ± 183.6	487.1 ± 112.6	<0.001
Age (years)	57.5 ± 4.3	57.7 ± 4.3	0.121
Women (%)	57.8	44.2	<0.001
Supine SBP (mmHg)	122.0 ± 16.3	124.7 ± 16.8	<0.001
Supine DBP (mmHg)	76.0 ± 9.6	78.0 ± 9.9	<0.001
ΔSBP (mmHg)	−3.6 ± 10.2	−4.3 ± 10.5	0.026
ΔDBP (mmHg)	−9.5 ± 6.4	−9.4 ± 6.6	0.421
Supine HR (beats/minute)	60.4 ± 8.8	62.3 ± 9.4a	<0.001
Orthostatic hypotension (%)	2.4	1.9	0.246
Orthostatic hypertension (%)	5.2	6.8	0.024
Current smoking (%)	16.0	18.4	0.037
Hypertension (%)	21.7	31.1	<0.001
Diabetes/prediabetes (%)	19.7b	25.1a	<0.001
BMI (kg/m2)	26.6 ± 4.2	28.6 ± 5.0	<0.001
LDL (mmol/l)	3.6 ± 0.9 d	3.6 ± 1.0c	0.028
CACS (median [Q1-Q3])	0 (0–24.0)	0 (0–38.3)	<0.001*
CACS > 0 (%)	41.8	48.8	<0.001

Continuous variables are expressed as mean ± standard deviation, except for coronary artery calcium score which is expressed as median (Q1- Q3). Dichotomous data are expressed as percentages of total within each group. P-values denote overall differences from independent samples t-test for continuous data and Pearson’s chi2-test for dichotomous data unless otherwise indicated. A potential “at-risk” joint behaviour of sedentary and VPA, as well as sedentary and MPA, was defined as a high daily sedentary time (>9.5 h/day) and low VPA (<75 min/week) or low MPA (<150 min/week), respectively. Missing data: a=1, b=2, c=10, d=12. Data on smoking habits, hypertension and coronary artery score were missing in 139, 175 and 170 participants respectively. *Q* Quartile, *HR* heart rate, *SBP* systolic blood pressure, *DBP* diastolic blood pressure, *BMI* body mass index, *LDL* low-density lipoprotein, *CACS* coronary artery calcium score.

*=Mann-Whitney test.

Patients with a lower degree of MVPA had higher supine SBP and DBP. Delta SBP was greater among patients with low degree of MVPA whereas delta DBP was lower among patients with low degree of MVPA. Manifest OH was more prevalent among patients with the highest amount of MVPA (*p* = 0.002). Participants with manifest OHTN were more likely to have an “at-risk” PA pattern and a lower level of MVPA (*p* = 0.024 and 0.002 respectively). In contrast, OHTN was not associated with a high proportion of sedentary time (*p* = 0.098).

### Association between blood pressure during orthostatic tests and physical activity

**Fig. 2:**
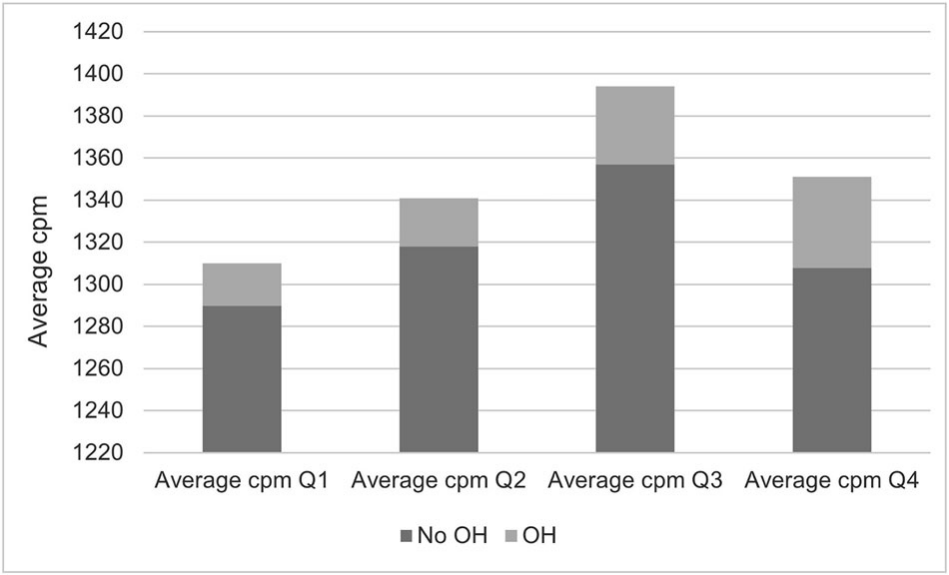
Orthostatic hypotension according to physical activity status. Percentage of participants with orthostatic hypotension (light grey) or no orthostatic hypotension (dark grey) according to average physical activity intensity groups (Quartiles[Q] 1-4). Q1 indicates the least active individuals whereas Q4 indicates the most active individuals. Cpm counts per minute, OH orthostatic hypotension.

In linear regression models, lower sedentary time or higher MVPA and higher average cpm were associated with a greater decrease in delta SBP from baseline to 3 min after adjusting for age, sex, diabetes, smoking, pulse, total wear time, proportion weekend days, and season (*p* < 0.05 for all). A higher time spent sedentary or lower MVPA was associated with higher SBP and DBP after 3 min of active standing (*p* < 0.01) for all after adjusting for age, sex, total wear time, proportion weekend days and season. The significant association between MVPA and DBP after 3 min disappeared when adjusting for age, sex, diabetes, smoking, pulse, total wear time, proportion weekend days, and season (*p* = 0.070), however. Delta DBP was not associated with level of sedentary time, although there was a negative significant association between delta DBP and higher MVPA and average cpm when adjusting for age, sex, total wear time, proportion weekend days, and season (*p* = 0.017 and *p* = 0.037, respectively) (Table 6). The proportion of individuals who met criteria for OH was greater among individuals who were more physically active (*p* = 0.011, Fig. 2).

**Table 6:** Association between physical activity and orthostatic blood pressure among middle aged men and women.

	Dependant variable	Beta	P1	P2
Sedentary time (%)	SBP 3 min	0.089	<0.001	0.007
Moderate-vigorous Physical activity (%)	SBP 3 min	−0.405	<0.001	<0.001
Average counts per min	SBP 3 min	−0.007	<0.001	<0.001
Sedentary time (%)	ΔSBP	−0.044	0.001	0.003
Moderate-vigorous Physcial activity (%)	ΔSBP	0.124	0.003	0.010
Average counts per min	ΔSBP	0.002	<0.001	0.002
Sedentary time (%)	DBP 3 min	0.068	<0.001	<0.001
Moderate-vigorous physical activity (%)	DBP 3 min	−0.142	<0.001	0.070
Average counts per min	DBP 3 min	−0.003	<0.001	0.001
Sedentary time (%)	ΔDBP	0.003	0.682	0.927
Moderate-vigorous physical activity (%)	ΔDBP	−0.061	0.017	0.036
Average counts per min	ΔDBP	−0.001	0.037	0.075

Multivariable-adjusted linear regression models including percentage sedentary PA, MVPA or average counts per minute as independent variable and SBP or DBP as dependant variable (either delta SBP/DBP or SBP/DBP after 3 min of standing). Delta SBP/DBP were defined as supine blood pressure minus blood pressure value after 3 min of active standing. *SBP* systolic blood pressure, *DBP* diastolic blood pressure.

P1 adjusted for age, sex “Wear Time Total”, “Weekend Days Percentage” and “Season”.

P2 adjusted for age, sex, diabetes/prediabetes, current smoking, supine heart rate, “Wear Time Total”, “Weekend Days Percentage” and “Season”.

### Association between orthostatic hypotension and coronary artery calcium score and interaction effects of physical activity

There was a significant association between CACS and OH. Individuals with CACS > 0 had a greater prevalence of OH compared to participants with CACS ≤ 0 (n [%]; 62 [2.7] versus 55 [1.9]; *p* = 0.041). In contrast, individuals with CACS > 0 and CACS ≤ 0 had similar proportions of OHTN (n [%]; 138 [6.0] versus 157 [5.3]; *p* = 0.276). We did not find any significant interaction effects of PA (% sedentary time, MVPA or “at risk”) on the association between orthostatic BP reactions (delta SBP, delta DBP, OH, OHTN) and CACS (data not shown).

## Discussion

We report that the level of PA assessed by accelerometery in middle-aged subjects was associated with BP reactions upon standing. Physically active individuals had lower increase in SBP and a greater increase in DBP during active standing compared to less active individuals. Somewhat unexpectedly, manifest OH was more prevalent among physically active participants whereas OHTN was more prevalent among less physically active individuals. Increased subclinical coronary atherosclerosis was associated with a higher prevalence of OH, but not OHTN. We did not find any interaction effects of PA on the association between subclinical coronary atherosclerosis and orthostatic BP reaction.

### Orthostatic hypotension, orthostatic hypertension, and physical activity

A healthy response to standing is characterized by no change, or a limited reduction in SBP but an increased DBP, resulting in a narrowing of the pulse pressure [18]. In the current study, participants with a high proportion of sedentary time or a low MVPA had a greater increase in SBP, but a lower increase in DBP compared to more active individuals. This suggests that physically inactive individuals have a less favourable hemodynamic response to standing. Previous studies support this, linking higher cardiorespiratory fitness and PA to better cardiovascular autonomic function due to higher cardiac vagal and lower sympathetic activity [19, 20]. The proportion of participants with manifest OHTN was higher among less physically active individuals. OHTN has been less studied than OH, however increasing data suggest that OHTN is a marker of cardiovascular autonomic instability and an important risk marker for subclinical CVD [4].

The proportion of participants with manifest OH was higher among more active individuals. Physically active individuals had a lower baseline SBP and consequently had lower orthostatic SBP. However, one might speculate that PA, with possible effects on systemic vasodilatation, might worsen BP instability among individuals predisposed for OH. On the other hand, exercise of leg and abdominal muscles is suggested as additional therapy for OH patients [1], although its efficacy is less well established. Previous studies suggest that an acute bout of exercise exacerbates OH in the short term in middle-aged individuals with chronic autonomic failure [21]. Furthermore, an 8-week home-based resistance training program did not improve orthostatic BP in older adults with OH, even though symptoms improved [22]. Previous studies found that regular PA increases bone and muscle strength as well as postural control in older patients [23]. Exercise is also associated with greater functional ability and decreased prevalence in falls among older individuals [24]. Although our current results do not seem to support the recommendation of PA as an important lifestyle advice for individuals with OH, it must be emphasized that the prevalence of this condition was very low (around 2%) in this generally healthy middle-aged population. One might speculate that the few subjects with manifest OH, who also had more risk factors, had been recommended to be more physically active, suggesting that the higher PA registered among those individuals might be a consequence rather than a cause of their condition. Thus, we cannot draw any conclusions on how PA may influence orthostatic BP reaction in OH patients.

### Pathophysiological mechanisms behind the relationship between physical activity and orthostatic blood pressure reactions

It has been suggested that loading of arterial baroreceptors may be required to maintain normal baroreflex function [25]. Prolonged exposure to bedrest and physical inactivity has been suggested to remove baroreceptor unloading caused by regular upright standing and causes attenuation of cardiovascular baroreflex responses, which may provoke OH 25]. In contrast, exercise has been shown to increase arterial baroreceptor loading and cause an acute increase in carotid baroreceptor sensitivity which was associated with enhanced orthostatic stability [26]. Furthermore, a previous study found that cardiorespiratory fitness was positively associated with immediate vagal withdrawal, which potentially diminished acute decrease in orthostatic BP. This immediate vagal withdrawal was followed by efficient vagal rebound, possibly due to increased baroreflex sensitivity with cardiorespiratory fitness [27]. On the other hand, it has been suggested that endurance-trained athletes may have reduced orthostatic tolerance [28]. The researchers speculated that increased leg compliance and venous pooling in lower extremities, ventricular hypertrophy, and a reduction in carotid and aortic baroreflex responsiveness may explain this [28]. The reason for the contrasting results may be due to different study populations, in the latter case including trained athletes.

### Subclinical coronary atherosclerosis, physical activity and orthostatic blood pressure reaction

It is unclear if an abnormal orthostatic BP reaction is a cause or a consequence of (subclinical) CVD. OH has been associated with arterial stiffness [29] and activated systematic inflammation [30], which are both involved in the pathogenesis of CVD [31]. Also, dysfunction in the baroreflex, probably induced by impairment of the baroreceptor due to aging or atherosclerosis is one of the most important causes of OH [32]. Furthermore, baroreflex dysfunction has recently been associated with the severity of subclinical coronary atherosclerosis, suggesting that baroreflex dysfunction may be involved in the early process of coronary atherosclerosis [33]. In a recent study performed in a young to middle aged Swedish population, both higher and lower BP upon standing was associated with increased arterial stiffness [34].

Few studies have investigated if PA modifies the relationship between autonomic dysfunction and CVD. PA could be a potential confounding factor, affecting both the autonomic nervous system and CVD [6, 35]. A previous study reported that metabolic syndrome components were negatively associated with HRV indices and higher HR, whereas PA was associated with higher HRV and lower HR [35]. Our hypothesis was that PA could diminish the association between cardiovascular autonomic dysfunction and coronary atherosclerosis. OH was more prevalent among participants with a higher CACS, corroborating findings from previous studies on coronary artery disease and OH [3, 36]. However, we did not find any significant interaction effects of PA and the association between subclinical coronary atherosclerosis, PA and orthostatic BP reaction, possibly due to the study population which consisted of middle aged and predominantly healthy individuals.

### Strengths and limitations

The strengths of the present study include a likely representative sample from a middle-aged study population, a relatively high participation rate, a low dropout rate and a high proportion of participants with valid accelerometer data. Accelerometers used to assess the PA pattern give a more valid estimate of actual daily movement patterns than self-reported data. The current study also has some limitations. Firstly, hi*p*-worn accelerometers convey risks of underestimating PA during cycling [37], water based activities, and upper limb activities. Secondly, this study included predominantly healthy and relatively young participants, which may explain why we did not find any significant interaction effects of PA patterns on BP reaction and subclinical coronary atherosclerosis. Future studies should further investigate potential interaction effects among older patients. Thirdly, the sample presented a narrow age range of middle-aged men and women, limiting the generalizability of the findings to younger or older individuals. Lastly, there is no consensus on cut-off values for accelerometery data. However, in the current study, sedentary and PA cut-offs were chosen according to most previous studies to enable comparisons with previous research [11–13].

## Conclusion

Individuals that are more physically active have a smaller increase in systolic BP and a greater increase in diastolic BP during active standing compared to less active individuals. This indicates that physical activity may be associated with a healthier orthostatic BP reaction. On the other hand, the prevalence of excessive BP decrease during standing was higher among individuals with high levels of physical activity. Furthermore, PA did not modify the association between orthostatic BP-response and subclinical atherosclerosis. The potential consequences of these findings are unclear and warrants further research in cohorts with a higher prevalence of manifest orthostatic hypotension.

## Summary

### What is known about the topic?

- An abnormal blood pressure (BP) response on standing is associated with atherosclerotic cardiovascular disease (CVD).
- Physical activity (PA) may improve cardiovascular autonomic function and reduce the risk of CVD.
- However, the role of PA on orthostatic BP-reactions and its relation to subclinical atherosclerosis is unclear.

### What this study adds?

- Higher PA levels were linked to a smaller systolic and greater diastolic BP increase during standing, suggesting a healthier response. However, excessive BP decreases during standing were more common in highly active individuals.
- PA did not modify the association between orthostatic BP-response and subclinical atherosclerosis.

## Supplementary information

Supplementary table 1

## Data Availability

Additional data are available from the corresponding author on reasonable request.
