# Supplementary material for: The relationship between physical activity, orthostatic blood pressure reactions and subclinical atherosclerosis: the Swedish CArdioPulmonary bioImage Study (SCAPIS)
Source: J Hum Hypertens. 2025 May 5;39(6):392–9. doi: 10.1038/s41371-025-01022-8 (PMC12151865; doi:10.1038/s41371-025-01022-8)
Supplement: Supplementary file 1 — Supplementary table 1 [file 41371_2025_1022_MOESM1_ESM.docx]

**Supplementary table 1.** Baseline characteristics of participants according to sex.

| Characteristic (total n=5396) | Women | Men | p-value |
| --- | --- | --- | --- |
| Age (years) | 57.4±4.2 | 57.7±4.4 | 0.049 |
| Current smoker (%) | 16.7 | 16.6 | 0.948* |
| Hypertension (%) | 23.2 | 25.6 | 0.047** |
| Diabetes/prediabetes (%) | 17.7 | 25.2 | <0.001*** |
| Supine HR (beats/minute) | 61.5±8.7 | 60.2±9.4 | <0.001 |
| Supine SBP (mmHg) | 120.3±17.1 | 125.6±15.2 | <0.001 |
| Supine DBP (mmHg) | 75.6±9.7 | 77.6±9.6 | <0.001 |
| ΔSBP (mmHg) | -4.4±10.4 | -3.1±10.2 | <0.001 |
| ΔDBP (mmHg) | -9.6±6.4 | -9.4±6.6 | 0.304 |
| CACS (median (Q1-Q3)) | 0(0-4) | 5(0-75) | <0.001†**** |

_Continuous variables are expressed as mean±standard deviation, except for coronary artery calcium score which is expressed as median (Q1- Q3). Dichotomous data are presented as percentages within each group. P-values denote overall differences from independent samples t-test for continuous data and Pearson’s chi2-test for dichotomous data unless otherwise indicated._

_Missing data: *=136, **=172, ***=3, ****=170. Q= Quartile, HR=heart rate, SBP=systolic blood pressure, DBP=diastolic blood pressure, CACS = coronary artery calcium score, bpm=beats per minute._

_† p-value from Mann-Whitney U test._
